# Histones, Their Variants and Post-translational Modifications in Zebrafish Development

**DOI:** 10.3389/fcell.2020.00456

**Published:** 2020-06-05

**Authors:** Vincenzo Cavalieri

**Affiliations:** ^1^Laboratory of Molecular Biology and Functional Genomics, Department of Biological, Chemical and Pharmaceutical Sciences and Technologies (STEBICEF), University of Palermo, Palermo, Italy; ^2^Zebrafish Laboratory, Advanced Technologies Network (ATeN) Center, University of Palermo, Palermo, Italy

**Keywords:** histone, histone posttranslational modifications, histone variants, epigenetics, development, maternal-to-zygotic transition, zygotic genome activation, zebrafish

## Abstract

Complex multi-cellular organisms are shaped starting from a single-celled zygote, owing to elaborate developmental programs. These programs involve several layers of regulation to orchestrate the establishment of progressively diverging cell type-specific gene expression patterns. In this scenario, epigenetic modifications of chromatin are central in influencing spatiotemporal patterns of gene transcription. In fact, it is generally recognized that epigenetic changes of chromatin states impact on the accessibility of genomic DNA to regulatory proteins. Several lines of evidence highlighted that zebrafish is an excellent vertebrate model for research purposes in the field of developmental epigenetics. In this review, I focus on the dynamic roles recently emerged for histone post-translational modifications (PTMs), histone modifying enzymes, histone variants and histone themselves in the coordination between the precise execution of transcriptional programs and developmental progression in zebrafish. In particular, I first outline a synopsis of the current state of knowledge in this field during early embryogenesis. Then, I present a survey of histone-based epigenetic mechanisms occurring throughout morphogenesis, with a stronger emphasis on cardiac formation. Undoubtedly, the issues addressed in this review take on particular importance in the emerging field of comparative biology of epigenetics, as well as in translational research.

## Introduction

The genomic information of eukaryotic cells is confined inside the nucleus in the form of chromatin, a nucleoprotein complex composed primarily of DNA and histone proteins, but also including noncoding RNA and a variety of structural non-histone proteins (Kornberg, 1974; Rodríguez-Campos and Azorín, 2007; Bonev and Cavalli, 2016). The basic repeating unit of this periodic structure, called the “nucleosome core particle,” consists of 147 base pairs of DNA wrapped nearly twice in a left-handed toroidal supercoil around a positively charged protein octamer containing two copies of each of four core histones H2A, H2B, H3, and H4 (Luger et al., 1997; Kornberg and Lorch, 1999). A fifth histone type, H1, interacts with the two internucleosomal linker DNA arms extending from a core particle, thus favoring the establishment of additional hierarchical levels of chromatin compaction (Zhou et al., 2013; Bednar et al., 2017).

Nucleosomes not only act as fundamental units of chromatin packaging, but also play pivotal roles in the coordination between chromatin architecture and functions by means of epigenetic mechanisms (Cavalieri et al., 2009). Among these, covalent post-translational modifications (PTMs) of specific amino acid residues on histones operate in combinatorial fashions either at a single nucleosome level or in a genome-wide manner, thus contributing to an extensive range of biological processes including organism development (Bhaumik et al., 2007; Lee et al., 2010; Cavalieri and Spinelli, 2015). More specifically, the presence of histone PTMs stereochemically alters the binding affinity of the nucleosomes for regulatory complexes that can be recruited or drawn away from chromatin (Smith and Shilatifard, 2010). Although modern molecular biology and mass spectrometry-based methods allowed the discovery of an ever-growing number of histone PTMs, acetylation (ac) and (mono-, di-, and tri-) methylation (me1, me2, and me3, respectively) of lysine (K) residues are the most thoroughly investigated (Di Caro et al., 2007; Zhao and Garcia, 2015; Janssen et al., 2017). Generally speaking, histone PTMs represent repositories of epigenetic memory over multiple generations, especially in those organisms lacking in conventional DNA methylation (Turner, 2009; Cavalieri and Spinelli, 2019). Nonetheless, histone PTMs are not permanent epigenetic marks, because an assorted group of histone-modifying enzymes dynamically governs the attachment or removal of small chemical groups on specific amino acid residues, thereby providing a valuable epigenetic mechanism of cellular adaptation in fluctuating environments (Kouzarides, 2007).

The physicochemical properties of nucleosomes can also be altered by exchanging conventional histone proteins with histone variants showing distinct amino acid sequences compared to their canonical counterparts (Weber and Henikoff, 2014). Such replacements may permit the specific nucleosome recognition, otherwise precluded, by chromatin modifying complexes that will successively appose variant-specific PTMs (Talbert and Henikoff, 2017). Nevertheless, histone variants are often subjected to the same PTMs as their canonical counterparts.

Cell fate decisions made during embryogenesis also depend upon the modulatory control of histone PTMs, histone variants and other epigenetic processes, on the hierarchical cascades of transcriptional events outlining the developmental gene regulatory networks of a given organism (Cavalieri et al., 2008, 2013; Balasubramanian et al., 2019; Horsfield, 2019). In particular, failures in establishing or maintaining proper restrictive and/or permissive patterns of histone PTMs, as well as alterations in histone variant deposition, can seriously disturb the developmental program (Bhaumik et al., 2007; Chen et al., 2013; Maze et al., 2014).

The small freshwater cyprinid *Danio rerio*, commonly known as zebrafish, offers unique opportunities to investigate histone epigenetic dynamics during vertebrate development. The increasing popularity of this model is due to two main reasons: (1) components of the epigenetic machinery have been widely characterized in zebrafish, showing overall conservation with mammals (Howe et al., 2013; Cavalieri and Spinelli, 2017), and (2) zebrafish embryos are optically translucent and relatively permeable to water-soluble compounds, allowing non-invasive live imaging of morphogenetic events and phenotypes following exposure to environmental stressors acting on the epigenome (Godinho, 2011; Ali et al., 2014). No less important benefits include ease of husbandry and maintenance in laboratory, high fecundity, external fertilization, short life cycle and generation time (Cavalieri and Spinelli, 2017).

## Multiple Coordinated Histone-Related Epigenetic Events Accompany Early Embryogenesis

**FIGURE 1:**
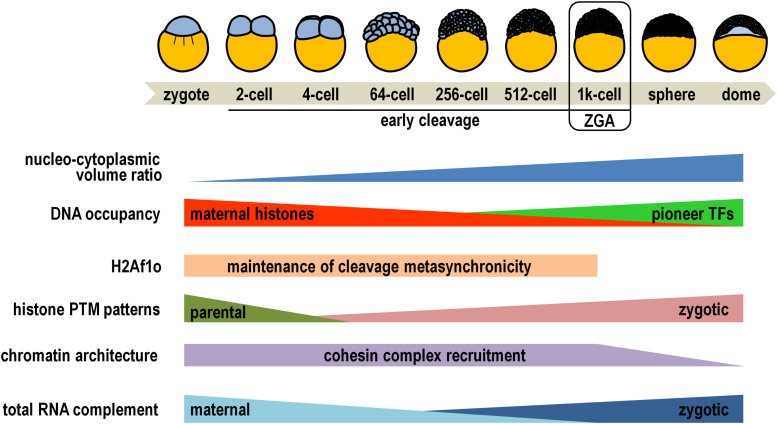
Diagrammatic representation of key epigenetic changes occurring during early zebrafish embryogenesis. Simplified drawings on the top depict some of the developmental stages, while developmental and epigenetic trends are illustrated below (see main text for details). Please note that placeholder nucleosomes retain parental histone modification patterns throughout early embryogenesis, and that cohesin complex recruitment is restored at 24 h post-fertilization. PTM, post-translational modification; TFs, transcription factors; ZGA, zygotic genome activation.

In zebrafish, core histones themselves play a first prominent role during the early cleavage phase of development (Figure 1), when multiple rounds of synchronous cell division convert the newly fertilized egg into a multicellular embryo composed of pluripotent blastomeres (Kimmel et al., 1995). At this time, the nascent embryonic genome is substantially, but not completely, inert in transcription due to dynamic competition for DNA binding between the stoichiometric excess of chromatin-unbound maternal histones and a small set of pioneer transcription factors that, later on, will determine the onset of major zygotic transcription (Lee et al., 2013; Heyn et al., 2014; Joseph et al., 2017). While this competition takes place, the oocyte-specific H2A variant H2Af1o is ubiquitously distributed in the early embryo, where it is critically required for maintaining blastomere cleavage metasynchronicity (Figure 1), probably by conferring a more relatively loose nucleosomal structure than canonical H2A (Kane and Kimmel, 1993; Olivier et al., 2010; Yue et al., 2013).

Within nuclei of cleaving zebrafish embryos, the epigenetic reprogramming process efficiently results in the rapid erasure of the bulk of parental histone PTMs from all nucleosomes except the so called “placeholder” nucleosomes (Figure 1). These specialized nucleosomes harbor the histone variant H2A.Z, termed H2AFV in zebrafish, and are decorated by H3K4me1 (Murphy et al., 2018). These features synergistically attenuate nucleosomal stability and prevent recruitment and/or activity of *de novo* DNA methyltransferases at promoter of housekeeping and regulatory genes (Lindeman et al., 2011; Hirano et al., 2019). Early developing embryos are loaded with maternal translationally competent mRNAs coding for a wide variety of histone methyltransferases and acetylases, comprehensively accomplishing renewal of zygotic-specific combinations of histone PTMs (Sun et al., 2008; Toyama et al., 2008; Aanes et al., 2011; Lindeman et al., 2011). These epigenetic signatures are overtly detectable at the promoter of about one thousand genes at the 256-cell stage and consist of H3K27ac enrichment restricted almost exclusively to placeholder nucleosomes, and combinations of H3K4me3, H3/H4ac (two PTMs associated with permissive chromatin), H3K27me3 and H3K9me3 (both associated with quiescent chromatin) on canonical nucleosomes (Zhang et al., 2018; Lindeman et al., 2010; Sato et al., 2019). For example, a typical profile foreshadowing high propensity for gene expression comprises co-occurrence of H3K4me3 and H3K9ac/H4ac, while simultaneous accumulation of H3K4me3 and H3K27me3 is reminiscent of bivalent promoters, considered to be in a poised transcriptional state ready for either rapid activation or permanent silencing following the maternal-to-zygotic transition (Puri et al., 2015).

Of not secondary importance, H3K4me3 marks directly correlate with the establishment of well-positioned nucleosome arrays on gene promoters, independently of robust RNA polymerase II binding, and prepare genes for subsequent transcriptional activation (Zhang et al., 2014). In fact, as the nuclear concentration of maternally supplied DNA-unbound histones drops following the progressive increase of the nucleo-cytoplasmic volume ratio at the mid-blastula transition stage (Figure 1), the three pioneer transcription factors Pou5f3, Nanog, and SoxB1, along with the chromatin remodeler Smarca4a, cooperatively finalize chromatin opening by sequential destabilization, displacement, and depletion of nucleosomes occupying the enhancers of developmental genes (Kane and Kimmel, 1993; Haberle et al., 2014; Joseph et al., 2017; Liu et al., 2018; Reisser et al., 2018; Veil et al., 2019).

In the standpoint of the three-dimensional genome architecture, gene-rich accessible chromatin conjointly exhibiting the mentioned permissive histone marks and occupancy of pioneer factors significantly coincides with cohesin complex recruitment (Figure 1), which is a prerequisite for spatial compartmentalization of the zebrafish genome before and during zygotic genome activation (Kaaij et al., 2018; Meier et al., 2018; Vallot and Tachibana, 2020).

Owing to such a strict hierarchical order of epigenetic developmental circuitries, the embryonic genome takes charge of gene expression and the maternal mRNAs are replaced coordinately in all blastomeres by zygotic gene products (Figure 1). Concertedly, the level of H3K4me3 increases further at promoter of transcriptionally active genes, and H3K36me3 (a mark associated with transcription elongation) specifically accumulates on their coding regions (Vastenhouw et al., 2010). By contrast, H3K27me3 enrichment extends dramatically throughout gene body of silenced loci, and heterochromatinization is outlined by removal of permissive PTM marks and concomitant increase in H3K9me3 levels (Lindeman et al., 2011; Laue et al., 2019).

## Morphogenesis Completion Engages a Multitude of Histone-Related Epigenetic Activities

Once the maternal-to-zygotic transition has been achieved, the embryo progresses toward gastrulation, during which the blastomere progeny begins to migrate and differentiate to give rise to distinct tissues and organs of the adult fish. Collectively, histone PTMs and their respective modifying enzymes/complexes play pivotal and surprisingly specific roles in ensuring that transcriptional states shift properly from pluripotent to cell type-specific patterns during morphogenesis. In particular, there is now mounting evidence that a multitude of histone lysine methyltransferases (HMTs)/demethylases (KDMs) and acetylases (HATs)/deacetylases (HDACs), as well as peculiar histone variants, are conjointly involved in epigenetic modulation of zebrafish organogenesis, and when their function is impaired, on a case by case basis, the embryo displays several types of organ malformations and/or dysfunctions (Table 1).

**TABLE 1:** Overview of studies examining the involvement of key histone modifying enzymes and histone variants in zebrafish development.

Epigenetic factors		Developmental processes	References
Histone modifiers	CMLO3	Axis elongation and head formation	Karmodiya et al., 2014
	HDAC1	Craniofacial development, neurogenesis, retinal differentiation, inner hear development, liver, and pancreas morphogenesis	Cunliffe, 2004; Stadler et al., 2005; Yamaguchi et al., 2005; Noël et al., 2008; Zhou et al., 2011; Ignatius et al., 2013; He et al., 2016a
	HDAC3	Liver and posterior lateral line development	Farooq et al., 2008; He et al., 2016b
	HDAC4	Perichondral ossification and pharyngeal skeleton development	DeLaurier et al., 2019
	HDAC5*	Cardiac valve formation	Just et al., 2011
	HDACs	Cardiac valve formation	Kim et al., 2012
	JMJD3	Myelopoiesis	Yu et al., 2018
	KAT2a and b	Craniofacial development	Sen et al., 2018
	KAT7	Angiogenesis	Yan et al., 2018
	KDM6ba	Brain, craniofacial, and heart development	Van Laarhoven et al., 2015; Akerberg et al., 2017
	KDM7	Brain development	Tsukada et al., 2010
	KMT2A	Neurogenesis	Huang et al., 2015
	KMT2D	Brain, craniofacial, and heart development	Van Laarhoven et al., 2015
	LSD1	Brain development Haematopoiesis	Li et al., 2012; Takeuchi et al., 2015
	MOZ	Pharyngeal segmentation	Miller et al., 2004
	PHF8	Brain and craniofacial development	Qi et al., 2010
	PRDM3 and 16	Craniofacial development	Shull et al., 2020
	PRMT1	Gastrulation movements	Tsai et al., 2011
	PRMT5	Germline differentiation	Zhu et al., 2019
	PRMT6	Gastrulation movements	Zhao et al., 2016
	SETDB2	Gastrulation movements	Du et al., 2014
	SET7/9	Myoblast differentiation	Tao et al., 2011
	SETD7	Heart morphogenesis	Kim et al., 2015
	SMYD3	Cardiac and skeletal muscle development	Fujii et al., 2011; Kim et al., 2015
	SMYD4	Heart morphogenesis	Xiao et al., 2018
	SMYD5	Haematopoiesis	Fujii et al., 2016
Histone variants	H2Af1o	Cell synchrony division before mid-blastula transition	Yue et al., 2013
	H2A.FV	Early embryogenesis	Sivasubbu et al., 2006; Madakashira et al., 2017; Murphy et al., 2018
	H2A.Z.2	Melanocyte differentiation	Raja et al., 2020
	H3.3	Cranial neural crest differentiation	Cox et al., 2012
	macroH2A1 and 2	Brain, somite, and fin development	Buschbeck et al., 2009; Gonzalez-Munoz et al., 2019

* Inactivation of HDAC5 is required during cardiac valve formation.

As a general rule, several epigenetic modifiers initially showing ubiquitous distribution in the early embryo undergo gradual restriction of their spatial expression pattern as development proceeds. In this scenario, one very pertinent example is provided by the interplay between distinct epigenetic modifications and mechanisms during cardiogenesis. More specifically, the SET and MYND domain-containing SMYD4 HMT becomes progressively restricted to the developing cardiovascular system, where it is directly involved in histone H3K4 di- and tri-methylation, and required to safeguard the proper acetylation level of histone H3 on the same chromatin targets, by recruitment and inactivation of HDAC1 (Xiao et al., 2018). Beyond being an obvious example of cross-talk between chromatin modulators, the SMYD4-HDAC1 association provides an excellent mechanism of tissue-specific regulation of the HDAC1 enzyme, otherwise operating throughout broad sectors of the developing embryo (Cunliffe, 2004; Pillai et al., 2004).

Global levels of H3K4me3 in the chromatin of the forming heart are defined by synergistic involvement of additional HMTs, including SMYD3 and the member of the SET domain-containing family SETD7. In fact, the mono-methyltransferase activity of SETD7 on naïve H3K4 is necessary for successive di- and tri-methylation by SMYD3 (Kim et al., 2015). KMT2D, another component of the SET domain-containing family of HMTs, is non-redundantly implicated in the establishment of H3K4me3 during the progression of cardiac looping (Van Laarhoven et al., 2015). Furthermore, KMT2D associates with KDM6A, a histone demethylase responsible for removal of the H3K27me3 repressive mark from cardiomyocyte chromatin (Issaeva et al., 2007; Van Laarhoven et al., 2015). In this role, the functional cooperation of additional H3K27-specific demethylases, including KDM6Ba and KDM6Bb, is required to promote cardiac trabecular outgrowth (Akerberg et al., 2017). However, this finding does not necessarily mean that H3K27 methylation is completely abolished in cardiomyocytes. Indeed, it should be emphasized that bulk H3K27me3 levels vary widely among different cardiac cell types, suggesting that distinct KDMs could deal with cell type-specific profiling of H3K27me3 through heart morphogenesis (Akerberg et al., 2017).

Although some studies indicated that differentiating cardiac cells necessitate physiological inactivation of specific HDACs, such as HDAC1 and HDAC5, exposure of developing embryos to chemical inhibitors for classes I and II HDACs revealed that general HDAC activity is critically required for the homeostatic balance of histone acetylation in the time window during which heart looping and cardiac valve formation occur (Huynh and McKinsey, 2006; Just et al., 2011; Kim et al., 2012; Xiao et al., 2018).

The combinatorial effect of all the mentioned epigenetic regulators eventually associates with the appropriate expression of cardiac marker genes (Fujii et al., 2011; Just et al., 2011; Kim et al., 2012; Xiao et al., 2018). Of interest, the epigenetic repertoire underlying chromatin of this set of genes typically comprehends elevated occupancy of the replacement histone variant H3.3, as revealed by means of a stable transgenic zebrafish line expressing a biotinylated version of H3.3 exclusively in cardiomyocytes (Goldman et al., 2017). Genome-wide profiling of H3.3-containing nucleosomes also revealed that enrichment of H3.3 alone is a reliable epigenetic indicator of enhancer activity within distinct cardiac subpopulations (Goldman et al., 2017). Conversely, the macroH2A2 histone variant is accumulated throughout the embryo body, in chromatin of both dividing and non-dividing cells, when heart formation processes take place (Buschbeck et al., 2009). It is worth noting, however, that hundreds of genes involved in morphogenesis of cardiac muscle and heart contraction map within chromatin regions enriched in macroH2A2, suggesting important roles for this histone variant (Gonzalez-Munoz et al., 2019). In principle, macroH2A2 occupancy largely coincides with both H3K27me3 and H3K9me3 heterochromatic marks. In spite of this, however, positive and negative mechanistic roles on the degree of chromatin accessibility could be equally postulated for this histone variant, since it is apparently associated with both repressing or activating transcriptional effects, depending on the cell type-specific chromatin context (Gonzalez-Munoz et al., 2019).

## Concluding Remarks and Further Perspectives

Cumulative findings argue against the idea that chromatin modifications, especially histone PTMs, could represent an instructive epigenetic code for switching on and off the transcriptional state of genes, as initially thought (Strahl and Allis, 2000; Henikoff and Shilatifard, 2011). Rather, deposition of histone variants and histone PTMs represent dynamic epigenetic features that either modulate chromatin accessibility through recruitment of chromatin remodeling machinery or are added as a consequence of gene transcription (Bartholomew, 2014).

It is clear that histone PTMs and histone variants have fundamental functions throughout zebrafish development, both in early totipotent blastomeres and nearly all differentiating cell types. Whereas the largest proportion of research studies in this field have described genome-wide patterns of histone PTMs and histone variant occupancy, understanding of direct mechanistic relationships between each of these epigenetic marks and specific loci involved in developmental processes is largely missing. On top of that, a lot of histone PTMs characterized in other organisms remain almost unexplored in zebrafish.

Combining characterization of chromatin modification dynamics on the transcriptional outcome of selected genomic regions with detailed phenotypic analysis of developing zebrafish requires advanced *in vivo* imaging techniques. For example, stated in simple terms, tracking particular types of histone PTMs in living cells requires a probe that specifically recognize them, once established, and a separate tag that allows visual identification of target-probe association. With this rationale, the “mintbody” methodology employs a single-chain variable fragment antibody fused to the enhanced green fluorescent protein to track residue-specific histone PTMs in living organisms, at a single cell level and on specific loci (Yao et al., 2006; Sato et al., 2013; Kimura et al., 2015). Remarkably, generation of viable and fertile transgenic zebrafish expressing a H3K9ac-specific mintbody demonstrated that this molecular tool does not significantly disturb normal cell functions, probably because it binds H3K9ac-containing nucleosomes for intermittent short time intervals, thus allowing regular access to chromatin complexes. Certainly, this powerful imaging analysis will greatly help our understanding of the relative contribution of histone PTMs on cell type-specific gene expression during zebrafish embryogenesis.

## Author Contributions

VC conceived the study, obtained the data, and wrote the manuscript.

## Conflict of Interest

The author declares that the research was conducted in the absence of any commercial or financial relationships that could be construed as a potential conflict of interest.
